# Factors affecting nurses' professional quality of life in Europe - A systematic review

**DOI:** 10.3934/publichealth.2026026

**Published:** 2026-04-16

**Authors:** Efrosini Vera, Petros Galanis, Polyxeni Mangoulia, Theodoros Pesiridis

**Affiliations:** 1 Faculty of Nursing, National and Kapodistrian University of Athens, 123 Papadiamantopoulou Str., Athens, 11527, Greece; 2 Clinical Epidemiology Laboratory, Faculty of Nursing, National and Kapodistrian University of Athens, 123 Papadiamantopoulou Str., Athens, 11527, Greece; 3 Community Nursing Laboratory, Faculty of Nursing, National and Kapodistrian University of Athens, 123 Papadiamantopoulou Str., Athens, 11527, Greece

**Keywords:** Europe, factors, nurses, nursing staff, professional quality of life

## Abstract

**Background:**

Nursing care involves both cognitive and emotional aspects, significantly impacting nurses' professional quality of life (ProQOL). This systematic review investigated the factors affecting nurses' professional quality of life in Europe.

**Methods:**

This systematic review followed the Preferred Reporting Items for Systematic Reviews and Meta-Analyses reporting framework and was registered in the International Prospective Register of Systematic Reviews (registration number: CRD420251133948). Searches were conducted in PubMed, Scopus, CINAHL, ScienceDirect, and EBSCOhost from inception to August 2025. Eligible peer-reviewed quantitative studies from European Union countries utilized the ProQOL instrument, reporting on all three dimensions: compassion satisfaction, burnout, and secondary traumatic stress. Two reviewers independently screened records, extracted data, and appraised methodological quality using the Joanna Briggs Institute critical appraisal tool.

**Results:**

A total of 3407 records were initially identified, and 35 studies were ultimately included, comprising 8580 nursing staff across diverse clinical settings, predominantly hospitals, with 13 studies conducted during the coronavirus disease period in 2019. Most studies reported moderate levels of compassion satisfaction and moderate levels of burnout and secondary traumatic stress. Higher compassion satisfaction was generally associated with greater age and professional experience, relevant education and training, work engagement, supportive work environments, better perceived control over workload, and stronger psychosocial resources, including resilience, psychological flexibility, self-compassion, mindfulness, and social support. Higher burnout and secondary traumatic stress related to indirect exposure were consistently linked to a high workload, overtime, rotating schedules, job stress, workplace violence, employment in high-intensity clinical areas, and adverse psychological states, including persistent stress, fear related to the coronavirus disease, and moral distress.

**Conclusions:**

Professional quality of life among European nursing staff is influenced by a combination of demographic, occupational, and psychosocial factors. Multilevel strategies addressing organizational stressors while strengthening individual and team-based resources are warranted to improve compassion satisfaction and reduce burnout and secondary traumatic stress.

## 1. Introduction

Healthcare workers encounter many occupational problems that render their work environment difficult and intricate [1]. Nurses, in particular, frequently endure the agony and pain of patients in their professional duties [2],[3]. In this case, caregivers have to deal with a lot of physical, emotional, and spiritual problems when they try to help and connect with patients and their families [1]. Nevertheless, the emotional burden is quite intense, and the relentless exposure to trauma can overwhelm even the most resilient nurses' capacity for self-regulation and coping. This leaves them increasingly vulnerable to psychological disturbances such as depression and anxiety [1]. They might use defense mechanisms to deal with stress and the demands of their jobs in response [4].

Nurses are the mainstay of health care delivery in public, private, voluntary, and educational sectors. Nevertheless, even though nurses make such a big contribution, they face many problems that influence their lives and careers. A poor professional status, small pay raises, a heavy workload, unpleasant working conditions, and low job satisfaction are some of the factors that affect their professional quality of life. Moreover, the persistent shortage of nursing staff, a situation worsened by the COVID-19 pandemic, has made the working circumstances for nurses progressively more difficult [5].

Stamm (2010), drawing on Figley's framework, conceptualizes the emotional life of professional caregivers as a dynamic balance between positive and negative outcomes of caring. Within this ProQOL model, professional quality of life reflects both the rewarding and costly aspects of providing care. Compassion satisfaction captures the pleasure, meaning, and fulfillment derived from helping others and performing one's job well, whereas burnout and secondary traumatic stress (STS) represent the negative consequences of chronic occupational stress and repeated exposure to others' trauma. Compassion fatigue is understood as the combined effect of burnout and STS, emphasizing that various individual, work-related, and contextual factors may operate either as protective factors that enhance compassion satisfaction or as risk factors that increase vulnerability to burnout and STS [6],[7]. The Professional Quality of Life Scale assesses these three dimensions and is widely used to examine how such factors shift the balance between positive and negative outcomes of caring [6].

The term “compassion fatigue” was first used by Joinson in Nursing Magazine in 1992 and describes symptoms that are similar to those of post-traumatic stress disorder (PTSD) [7]. The concept of compassion fatigue describes the negative effects of caring for sick or injured people. However, healthcare workers must bear the “cost of caring” when they are constantly surrounded by individuals experiencing pain and suffering [8]. Compassion fatigue refers to the detrimental effects of sustained empathetic involvement with suffering. Consequently, compassion may be considered a key part of the main ethical principles of nursing, and it is what drives nurses to provide great care [9].

Numerous primary research and systematic reviews, including meta-analyses, examine the correlation between professional quality of life and numerous contributing factors. Most of these studies, however, have been conducted in Asia, the USA, and other non-European countries, with a limited number focusing on the European context [10]–[22]. To date, no systematic review has specifically synthesized the factors associated with ProQOL among nurses working in the European Union countries. This focus is important because nurses' professional quality of life is shaped by healthcare system organization, staffing models, labor conditions, occupational demands, and broader social policy environments, all of which may differ substantially between European and non-European contexts. In addition, meaningful variation exists within Europe itself, as health services, resource allocation, and clinical working environments differ across countries and care settings. A Europe-specific synthesis is therefore needed to identify context-relevant determinants of nurses' professional quality of life and to better inform policy, workforce support, and organizational interventions within European healthcare systems.

The objective of this review was to examine the factors associated with professional quality of life among European nursing staff through a systematic review of the literature.

## 2. Materials and methods

### 2.1. Data sources and strategy

We conducted this systematic review in accordance with the Preferred Reporting Items for Systematic Reviews and Meta-Analyses (PRISMA) guidelines. We searched PubMed, Science Direct, Scopus, CINAHL, and EBSCOhost as databases from database inception to August 2025. The electronic database search was conducted between August 1–7, 2025. For the electronic search, we used combinations of terms related to nursing personnel, determinants, and professional quality of life. In titles and abstracts, the following search strategy was applied: (nurs* OR “nursing staff” OR “healthcare professionals” OR “health care professionals” OR “healthcare workers”) AND (factors OR predictors OR “risk factors” OR “independent variables”) AND (“professional quality of life” OR “work quality of life” OR “job quality of life”). The review protocol was registered with PROSPERO (CRD420251133948).

### 2.2. Selection and eligibility criteria

Two independent reviewers performed the study selection, and disagreements were resolved by a senior reviewer. After removing duplicates, titles and abstracts were screened, followed by full-text assessment of potentially eligible articles.

Studies were included if they: (a) involved nursing personnel at any professional level (e.g., registered nurses, nurse assistants); (b) were conducted in a European Union country; (c) were quantitative studies published in peer-reviewed scientific journals; and (d) used the Professional Quality of Life (ProQOL) instrument and examined all three dimensions of professional quality of life (compassion satisfaction, burnout, and secondary traumatic stress) and their associated factors.

Studies were excluded if they were not directly relevant to the research question of this systematic review. Case studies, editorial articles, reviews, study protocols, literature reviews, meta-analyses, qualitative studies, and mixed-methods research were also excluded. In addition, studies published in languages other than English, and studies involving nursing students, retired nurses, or nursing personnel working outside the European Union, were not considered eligible. Finally, studies that did not employ the Professional Quality of Life (ProQOL) instrument, or that did not assess all three dimensions of professional quality of life and their influencing factors, were excluded from the review.

### 2.3. Data extraction

Two independent authors performed the data extraction procedure, while a third author resolved discrepancies. A standardized data extraction form was used to ensure consistency across studies. From each included study, we extracted: authors, country, period of data collection, percentage of female participants, age, sample size, study design, sampling method, clinical setting, ProQOL version and assessment tools, response rate, and the factors examined in relation to each ProQOL dimension.

### 2.4. Quality assessment

The methodological quality of each included study was evaluated regarding validity, reliability, and consistency using the Joanna Briggs Institute (JBI) critical appraisal checklists for cross-sectional studies. The JBI checklist comprises 8 items for cross-sectional studies. Each item is rated as Yes, No, Unclear, or Not Applicable. Two reviewers independently appraised the studies, and disagreements were resolved through discussion or consultation with a third reviewer. Based on the proportion of items rated “Yes”, studies were categorized as: high quality (if 80% or more of the items were rated “Yes”), moderate quality (if more than 60% were rated “Yes”), and low quality (if fewer than 60% of the items were rated “Yes”) [23]. Potential risks of reporting bias were considered qualitatively in the interpretation of the findings.

### 2.5. Data synthesis and analysis

The included studies were grouped for synthesis according to key characteristics such as nurse specialty and geographic region, in order to facilitate comparisons across similar samples and contexts. Studies were included in each synthesis subgroup if they met all eligibility criteria described in Section 2.2.

All data were used as reported in the original studies. When information was incomplete or ambiguous, clarifications were derived from the article's context and, where necessary, through reviewer discussion and consensus. There was no contact with the study authors, and no imputation procedures were applied.

Given the heterogeneity in study designs, populations, exposures, and reported outcomes, a meta-analysis cannot be performed. Findings were interpreted in relation to study quality, geographic location, and population characteristics.

## 3. Results

### 3.1. Identification and selection of studies

**Figure 1.**
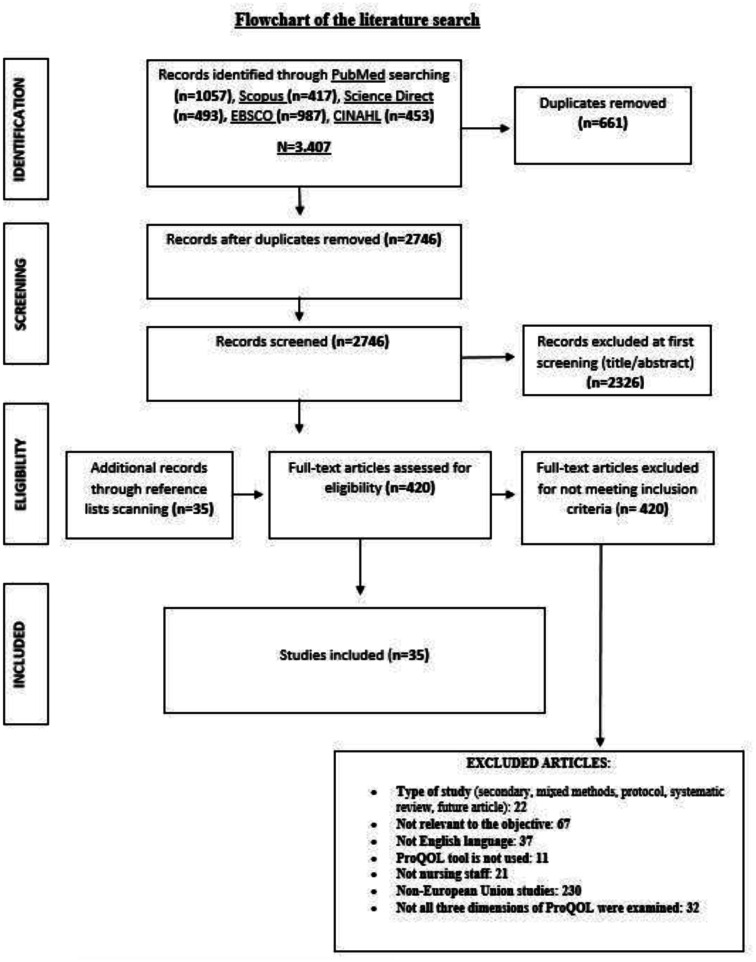
Flowchart of the systematic review [56].

A total of 3407 records were initially identified. After removing 661 duplicates, 2746 records remained for title and abstract screening, of which 2326 were excluded. Subsequently, 420 full-text articles were assessed for eligibility, and an additional 35 records were identified through reference list screening, resulting in 455 full-text articles being evaluated. Out of these, 420 articles were excluded after applying the inclusion and exclusion criteria. Ultimately, 35 studies were included in the review [1],[4],[7],[8],[24]–[55]. The flowchart of the literature search is presented in Figure 1.

### 3.2. Characteristics of the studies

Across the 35 studies included in this review, 8403 nurses and 177 nursing assistants were identified. The majority of the studies were conducted in Spain. In particular, thirteen studies were conducted in Spain [1],[4],[8],[24],[34],[36],[45],[48]–[52], nine in Greece [7],[38],[39],[41]–[43],[47],[54],[55], five in Portugal [27],[31],[32],[35],[53], four in Italy [28],[30],[33],[44], one in Poland [46], one in the Netherlands [40], one in Sweden [26], and one in Ireland [37]. This distribution indicates that the available evidence is concentrated mainly in Southern Europe, particularly Spain, Greece, Portugal, and Italy, whereas Northern and Central/Eastern European countries are represented by only a small number of studies. Therefore, the current evidence base provides only limited support for direct comparisons across European subregions. In 33 studies, female participants predominated. One study did not specify the gender distribution [51], and another reported a greater proportion of males (52%) than females [44]. Convenience sampling was used in all studies except two, one of which applied snowball sampling [53] and the other purposive sampling [47]. Among the included studies, thirty-two were cross-sectional, one was repeated cross-sectional, one was a three-wave prospective longitudinal study, and one was a retrospective cohort study. The nursing staff included in the studies worked in various healthcare settings, such as hospitals, primary care, neonatal and adult intensive care units, cancer centers, COVID-19 units, mental health centers, psychiatric residential units, day hospitals and day centers, long-term care facilities, rehabilitation centers, nursing homes, palliative care settings, structures of the Greek Red Cross, and obstetrics-gynecology services. It was observed that thirteen out of the thirty-five studies were conducted during the COVID-19 period. All studies used a validated tool to measure the professional quality of life (ProQOL scale).

The ProQOL scale includes three dimensions: compassion satisfaction, burnout, and secondary traumatic stress. Higher compassion satisfaction is associated with a higher professional quality of life, whereas increased burnout and secondary traumatic stress are indicative of a lower professional quality of life [6]. Overall, most of the studies reported moderate mean levels of compassion satisfaction and of the negative ProQOL dimensions (burnout and secondary traumatic stress). Reported response rates ranged from 12.20% to 96.6%, while seventeen studies did not specify their response rate. The main characteristics of the 35 included studies are presented in Table 1.

**Table 1.** Main characteristics of studies included in this systematic review.

Reference	Country	Data Collection Time	Females (%)	Age, Mean (SD)	Sample Size (n)	Study Design	Workplace	Response Rate (%)	Level of Analysis
[51]	Spain	NR	NR	NR	128 palliative care nurses, 16 nursing assistants	cross-sectional	palliative care setting	33.07%	High
[4]	Spain	2015	86.20%	39.8 (SD = 11)	297 oncology nurses	cross-sectional	hospital	81.50%	High
[50]	Spain	2020	76.70%	46.7 (SD = 10.2)	398 nurses	cross-sectional	healthcare services	NR	High
[25]	Spain	2018–2019	88.20%	18–65	69 nurses	cross-sectional	hospital	NR	High
[1]	Spain	2018	75.50%	47.32 (SD = 8.44)	1521 nurses (55% working in hospitals, 45% working in primary care)	cross-sectional	12 hospitals, 26 primary care districts, and 10 healthcare management areas (including primary care and hospital care)	NR	High
[8]	Spain	2018	84.50%	40.24 (SD = 9.78)	210 nurses	cross-sectional	healthcare public system	NR	High
[49]	Spain	2020	76.70%	46.7 (SD = 10.2)	398 nurses	cross-sectional	primary care, inpatient care, nursing homes, and specific COVID-19 units	NR	High
[52]	Spain	2020	91%	36.8 (SD = 5.4)	291 nurses	cross-sectional	nursing homes	96.60%	High
[48]	Spain	2018	78.30%	43.58 (SD = 7.68)	253 nurses	cross-sectional	hospitals	NR	High
[34]	Spain	2020	77.40%	43.9 (SD = 10.15)	129 nurses, 13 nursing assistants	cross-sectional	palliative care (not necessarily in PC settings)	87.57%	High
[45]	Spain	2019	85.30%	50–59	68 nurses	cross-sectional	general pediatrics, oncology, and PPC	91%	High
[36]	Spain	2020–2021	87.2% (2020)/ 87.8% (2021)	41.53 (2020)/ 41,44 (2021)	439 registered nurses in 2020 / 410 in 2021	repeated cross-sectional	hospitals, primary care centers, and long-term care facilities	NR	High
[24]	Spain	2019	90.30%	30–50	142 nurses, 18 nursing assistants	cross-sectional	obstetrics-gynecology service or neonatal intensive care unit	NR	High
[7]	Greece	2010–2011	70.10%	36.87 (SD = 7.37)	89 psychiatric nurses, 85 nursing assistants	cross-sectional	12 public hospitals	59%	High
[54]	Greece	2015	71.78%	33.5 (SD = 8.8)	44 nurses	cross-sectional	rehabilitation center	88.3%	High
[38]	Greece	2017–2018	78.80%	41.34 (SD = 8.3)	358 nurses	cross-sectional	five hospitals (in the adult and pediatric ICU, emergency, oncology, hematology, and neurosurgical departments, hemodialysis unit and operating theatre)	73.20%	High
[55]	Greece	2021	79%	38.27 (SD = 10.28)	33 nurses	cross-sectional	mental health centers, psychiatric residential units, day hospitals and day centers, psychiatric hospitals, and psychiatric departments of general hospitals	NR	High
[39]	Greece	2017	96.7%	37.3 (SD = 8.03)	121 nurses	cross-sectional	3 public hospitals	NR	High
[43]	Greece	2020	81.1%	37.2 (SD = 8.5)	29 nurses, 5 nursing assistants	cross-sectional	structures of the Greek Red Cross	NR	High
[41]	Greece	2020	75.3%	41.5 (SD = 10.4)	116 nurses	cross-sectional	hospital	78.80%	High
[47]	Greece	2021	73.5%	38.5	58 nurses	cross-sectional	hospitals	68%	High
[42]	Greece	2020	82.8%	42.3	258 ICU nurses	cross-sectional	hospitals for COVID-19	NR	High
[31]	Portugal	2014–2015	81.10%	37.66 (SD = 9.34)	280 nurses	cross-sectional	hospital	NR	High
[27]	Portugal	2017	65.50%	37.1 (SD = 6.3)	87 nurses	cross-sectional	emergency and urgent care unit for adults from a university hospital	94%	High
[32]	Portugal	NR	91.20%	39.06 (SD = 8.85)	221 oncology nurses	cross-sectional	hospital	76%	High
[53]	Portugal	2021	81.20%	37	586 nurses	cross-sectional	hospital	NR	High
[35]	Portugal	NR	84%	41	108 nurses	cross-sectional	oncology hospital	36%	High
[44]	Italy	2020–2022	48%	38 (SD = 8.1)	411 nurses	three-wave prospective longitudinal-design	hospital	86%	High
[28]	Italy	2021	62.2%	38.6	95 nurses	cross-sectional	hospital, working in the emergency department, pulmonology department, and hematology and bone marrow transplant centre	77.3%	High
[33]	Italy	2020	52.94%	46.6 (SD = 12)	24 nurses	cross-sectional	rehabilitation and psychiatric healthcare centers	69%	Moderate
[30]	Italy	2020	58.2%	42.29 (SD = 10.03)	122 nurses	cross-sectional	hospital	12.20%	High
[46]	Poland	2019–2020	96.7%	39 (SD = 11.51)	862 nurses	retrospective cohort study	hospital	NR	High
[37]	Ireland	NR	96%	36.59 (SD = 10.61)	60 nurses	cross-sectional	cancer care	NR	High
[26]	Sweden	2021	90%	25–45	71 nurses	cross-sectional	hospital	44%	High
[40]	Netherlands	2021	93.3%	45.2 (SD = 11.9)	215 nurses	cross-sectional	NICUs	NR	High

Note: SD: standard deviation; NR: not reported.

### 3.3. Quality assessment

The methodological quality of the included studies was assessed using the Joanna Briggs Institute (JBI) critical appraisal checklist for analytical cross-sectional studies. Based on the proportion of items rated “Yes” on the eight checklist questions, studies were classified as high quality (≥80% of items rated “Yes”), moderate quality (>60% of items rated “Yes”), or low quality (<60% of items rated “Yes”). Overall, thirty-four studies were rated as high quality [1],[4],[7],[24]–[32],[34]–[55] and one study was rated as of moderate quality [33]. No study was classified as low quality. It should be noted that one study, although described by the authors as a “retrospective cohort study,” was classified as cross-sectional in this review, as its methodology indicates that both exposure (perceived social support) and outcomes (compassion fatigue, burnout) were measured at a single point in time using self-reported questionnaires, and there was no follow-up period [46]. Therefore, for the purpose of this review, this study was classified and appraised as an analytical cross-sectional study. The detailed quality assessment of all 35 studies included in our review is presented in Supplementary Table S1.

### 3.4. Factors associated with ProQOL dimensions

#### 3.4.1. Demographic factors

Several demographic characteristics were consistently associated with higher levels of compassion satisfaction. In most studies, female nurses reported higher compassion satisfaction than their male counterparts [43],[50],[54]. Older age was also repeatedly linked to increased compassion satisfaction [1],[27],[38],[50],[55], suggesting that professional experience and emotional maturation may enhance the perceived rewards of caregiving. Marital and parental status further contributed to compassion satisfaction. Divorced or single nurses and those without children or with only one child tended to report higher compassion satisfaction [24],[41],[43],[53], possibly because they experience fewer competing demands between work and family roles. Overall, these findings indicate that demographic characteristics associated with greater life experience and fewer family responsibilities may facilitate the experience of compassion satisfaction among nurses.

In contrast, several demographic factors were associated with higher levels of burnout among nurses. Younger age and higher educational attainment emerged as risk factors for burnout [35],[38],[55], which may reflect unmet expectations and increased role strain at earlier career stages. Living and working in urban settings was also linked to higher burnout scores, possibly due to heavier caseloads and more complex care demands [1],[50].

Marital and parental status played a role, as well. Single nurses, particularly single women, more frequently reported emotional exhaustion and poorer health [28],[36],[49]. Being a parent was associated with increased difficulties in balancing work and family responsibilities and thus with greater burnout [35]. Taken together, these results suggest that younger, highly educated nurses with substantial family responsibilities and those working in urban environments may be especially vulnerable to burnout.

Similar patterns were observed for secondary traumatic stress and compassion fatigue. Female gender and younger age were repeatedly associated with higher levels of secondary traumatic stress [1],[7],[27],[28],[36],[50],[53],[55]. Working in urban areas was also linked to elevated compassion fatigue [1],[50].

Lower educational attainment and the absence of leisure activities were additional risk factors [27],[43], suggesting that fewer coping resources and limited opportunities for recovery outside work may exacerbate nurses' vulnerability to secondary traumatic stress. Overall, the demographic profile most consistently associated with compassion fatigue and secondary traumatic stress in this review was that of younger, female nurses with fewer coping resources and limited opportunities for rest and recreation.

#### 3.4.2. Work-related factors

Several occupational characteristics were consistently associated with higher levels of compassion satisfaction among nurses, thereby improving their professional quality of life. Specialized training in areas such as death, grief, or palliative care appeared to enhance nurses' sense of meaning and fulfilment at work, particularly in oncology and end-of-life care settings [45]. Longer work experience was also positively associated with compassion satisfaction in several studies [7],[26],[41],[49], suggesting that professional maturation and accumulated skills may help nurses derive greater reward from their caregiving role.

Certain employment and role characteristics further contributed to higher compassion satisfaction. Temporary employment contracts and a strong sense of identification with one's profession or care unit were linked to increased satisfaction in some studies [30],[45]. Nurses who reported a clear professional role, high levels of work engagement, and a positive, problem-oriented attitude were also more likely to experience compassion satisfaction [28],[47]. Working exclusively with children or with mixed populations that included pediatric patients was associated with higher compassion satisfaction compared with working solely with adults [38]. In addition, nurses and support staff tended to report higher compassion satisfaction than physicians in shared settings [38], and nurses who had personally chosen their unit or wished their children to pursue the same profession reported greater fulfilment [43].

Work setting and workload management also played an important role. Nurses working in emergency departments and experienced staff in high-acuity environments often reported higher compassion satisfaction, potentially because the impact of their interventions is more immediate and visible [41],[45]. Low levels of work–family conflict were positively associated with compassion satisfaction, indicating that a better balance between professional and personal life allows nurses to invest emotionally in their work without feeling overwhelmed [54]. Similarly, greater workload control and more effective coping with death were linked to higher compassion satisfaction and better overall ProQOL [34]. Taken together, these findings suggest that compassion satisfaction is fostered by a combination of adequate training, meaningful roles, supportive work environments, manageable workload, and congruence between personal and professional values.

A range of work-related factors increased the risk of burnout among nurses. Adverse work environments characterized by poor relationships with colleagues, lack of support, and generally negative organizational climates were consistently associated with higher burnout scores [38]. Exposure to workplace violence, including verbal and physical assaults by patients and relatives, further exacerbated emotional exhaustion and cynicism [7]. High workload, frequent overtime, and rotating shifts were also repeatedly identified as contributors to burnout [1],[25],[35],[38],[46],[49]. Temporary employment status and job insecurity emerged as additional stressors in some studies [49].

Working in highly demanding clinical areas, such as primary care, intensive care units, emergency departments, COVID-19 units, pathological wards, and palliative care services, was associated with increased burnout due to sustained exposure to complex clinical situations and high emotional demands [41],[50],[53]. Nurses with more than 20 years of experience, those who had not chosen their department and those considering leaving their unit were described as particularly vulnerable to emotional exhaustion and disengagement [4],[43],[49].

Job stress and its dimensions—including workload, time pressure, concerns about occupational safety, bureaucratic procedures, and role ambiguity—were repeatedly linked to higher burnout [35],[41],[53]. The lack of observable patient improvement despite intensive efforts was another source of frustration and emotional depletion [7]. Moreover, reliance on avoidance coping strategies was associated with higher burnout, whereas more adaptive coping appeared protective [28]. Overall, these findings point to a constellation of organizational stressors—heavy workload, shift work, role conflict, workplace violence, and lack of support—that collectively increase nurses' risk of burnout, especially in high-intensity settings [33],[38],[54].

Work-related determinants of compassion fatigue and secondary traumatic stress largely overlapped with those of burnout, but placed greater emphasis on exposure to trauma and emotionally charged situations. High workload, frequent overtime, and elevated job stress were consistently associated with higher secondary traumatic stress among nurses [25],[41],[46],[53]. Employment in high-stress environments such as COVID-19 units, emergency departments, and pathology services was particularly associated with compassion fatigue among nurses, assistant nurses, and physicians [1],[7],[38]. Non-ICU staff working in these settings sometimes reported higher levels of secondary traumatic stress, possibly due to fewer specialized resources and less training in managing acute critical incidents [38].

Persistent exposure to traumatic events, including caring for patients in intense suffering or terminal illness, was repeatedly described as a major risk factor for compassion fatigue [7],[35],[43]. Caring for women with traumatic birth experiences, unresolved loss histories, or complex psychosocial needs was associated with increased secondary traumatic stress among obstetric nurses [39]. Ongoing workplace violence, lack of patient improvement despite continuous care, and the cumulative intensity of traumatic experiences in healthcare settings further amplified psychological strain [7],[38].

Nurses who had not chosen their department or were considering leaving their unit appeared to be at particular risk of compassion fatigue, likely reflecting a misalignment between personal preferences and work demands [41],[50],[53]. The frequent use of avoidance coping strategies was also associated with higher secondary traumatic stress and did not mitigate the impact of repeated exposure to trauma [28]. Collectively, these findings suggest that compassion fatigue and secondary traumatic stress are shaped by the interaction of high-intensity clinical environments, repeated exposure to suffering and trauma, limited control over work assignments, and the use of less adaptive coping strategies.

#### 3.4.3. Psychological and social factors

A wide range of psychological and social resources emerged as important determinants of compassion satisfaction among nurses. Self-care practices, including physical, emotional, spiritual, and social self-care, were consistently associated with higher compassion satisfaction and better overall well-being [1],[44],[47],[51],[54]. Awareness and the ability to confront death, rather than avoid it, were also linked to higher compassion satisfaction among nurses working in palliative and end-of-life care [24],[51].

Psychological flexibility, resilience, and positive personality traits were particularly relevant. Higher levels of psychological flexibility and resilience were associated with greater compassion satisfaction and lower levels of burnout and secondary traumatic stress [25],[44],[49],[52]. Traits such as agreeableness, self-kindness, self-compassion, and self-acceptance were also positively related to compassion satisfaction, contributing to emotional balance and fulfilment in the caregiving role [25],[47],[49].

Interpersonal factors further supported compassion satisfaction. Empathic concern and perspective taking, when accompanied by adequate self-compassion and mindfulness, were associated with higher compassion satisfaction and lower distress [31],[32],[37],[44]. Perceived social support from colleagues, supervisors, family, and friends consistently emerged as a protective factor, buffering the impact of occupational stress and enhancing nurses' capacity to derive meaning from their work [28],[46],[49]. Mindfulness-based attributes, such as present-moment awareness and non-judgmental acceptance of internal experiences, were also positively associated with compassion satisfaction and negatively with burnout and secondary traumatic stress [31],[32],[37]. Overall, psychological resilience, self-compassion, mindfulness, and robust social support networks appear to be key resources that promote compassion satisfaction among nurses.

Conversely, several psychological and social factors were found to increase the risk of professional burnout among nurses. High levels of neuroticism and chronic stress were strongly associated with emotional exhaustion and reduced well-being at work [1],[25]. Frequent exposure to patients' suffering and traumatic events, as well as heightened fear related to crises such as the COVID-19 pandemic, intensified psychological strain and contributed to higher burnout scores [25],[36],[55]. Personal distress, self-judgement, and psychological inflexibility hinder adaptive coping and were repeatedly linked to burnout and secondary traumatic stress [31],[32],[37]. Social isolation and perceived lack of adequate social support exacerbated emotional fatigue and feelings of helplessness [28]. Additionally, emotional maladjustment and difficulties in regulating negative emotions further increased vulnerability to burnout [30]. Together, these findings underscore the importance of intrapersonal factors—such as personality traits, maladaptive cognitive emotional styles, and fear responses—as well as the availability of supportive interpersonal relationships in shaping nurses' susceptibility to burnout.

Several variables have been identified as contributing to heightened compassion fatigue among nurses. The absence of specialized training in managing emotionally challenging situations limited nurses' ability to cope effectively with repeated exposure to suffering and trauma. Neuroticism and openness were identified as personality traits associated with higher secondary traumatic stress. Neuroticism increased sensitivity to stress and negative effects, whereas openness might heighten emotional attunement to patients' suffering [25]. Avoidance behaviors, coupled with an intense fear of death, were also linked to greater emotional exhaustion and distress.

Frequent exposure to suffering and traumatic events, especially in high-intensity clinical areas, was a major risk factor for compassion fatigue, particularly when combined with high levels of job stress [7],[25],[35],[43]. Affective and cognitive empathy, while central to nursing practice, could contribute to emotional overload when not balanced by self-compassion and effective coping resources [7],[25],[31],[32]. Fear associated with the COVID-19 pandemic, moral distress arising from ethical dilemmas, empathic concern, and personal distress were additional psychological factors that depleted emotional resources and increased the likelihood of compassion fatigue [36],[42],[55].

Self-judgement, psychological rigidity, general psychological distress (stress, anxiety, and depression), and emotional maladjustment reduced resilience and impaired adaptive coping [30],[44]. These psychological and social determinants interact with demanding work contexts to heighten the risk of compassion fatigue and secondary traumatic stress, highlighting the need for interventions that strengthen psychological flexibility, resilience, self-compassion, and social support while reducing exposure to preventable stressors.

The detailed associations between specific factors and each ProQOL dimension are summarized in Table 2.

**Table 2.** Factors influencing nurses' ProQOL.

Reference	Demographic factors	Work-related factors	Psychological and social factors
[51]			↑CS: physical self-care, inner self-care, social self-care, awareness, coping with death
[4]		↑BO/↑STS: consideration to leave the unit	↑STS: not specialized training
[50]	↑CS: female, older age, being divorced ↑BO/↑STS: urban area, marital status	↑BO/↑STS: Primary care, Rotating shifts	
[25]	↑CS: age > 45 years	↑CS: training in death/ bereavement/palliative care ↑BO/↑STS: high workload	↑CS: resilience, agreeableness ↑BO: neuroticism, stress, exposure to suffering/ traumatic events ↑STS: neuroticism, openness (personality trait), avoidance, fear of death, exposure to suffering/traumatic events
[1]	↑CS: nursing profession ↑STS: female	↑BO: physicians ↑STS: physicians, working in COVID-19 units and in emergency departments	↑BO/↑STS: perceived stress
[8]			self-care and self-compassion increase ProQOL
[49]			↑CS: resilience, self-kindness ↓BO: resilience, isolation, over-identification, mindfulness ↑STS: affective empathy, cognitive empathy
[52]			↑CS: psychological flexibility
[48]	↑BO: single	↑CS: less work experience ↓CS: longer work experience, poor working conditions ↑BO/ ↑STS: temporary employment status, rotating shifts, >20 years of experience	↑CS: social support
[34]		↓ProQOL: work overload ↑ProQOL: workload control, coping with death	↑CS: self-compassion ↑ProQOL: awareness/self-compassion
[45]		↑CS: temporary employment contract, oncology nurses than hospital ward nurses ↑CF: longer work experience	↓CF: religious beliefs
[36]	↑BO/↑STS: female gender		↓CS: fear of COVID-19 ↑BO/↑STS: fear of COVID-19
[24]	↑CS: divorced		↑CS: training in coping with death
[7]	↑STS: years of experience, female	↑BO: exposure to patient assaults, ongoing workplace violence, lack of patient improvement ↑STS: assistant nurses, patients per unit, intention to leave, exposure to patient assaults, ongoing workplace violence, personal traumatic experiences	↑STS: personal traumatic experiences
[54]	↑CS: female	↑CS: low level of WFC (work/family conflict) ↑BO: nursing profession	↑CS: well-being
[38]	↑CS: older age, more years of experience ↑BO: younger age, high level of education	↑CS: working only with children or a mixed population of adults and children, nurses and support staff than doctors ↓CS: bad working environment, poor relations with colleagues, shifts, not working in a department of their choice ↑BO: working in ICU, heavy workload and/or lack of a supportive working environment, shifts, bad working environment, poor relations with colleagues, not working in a department by choice, doctors ↑STS: support staff, non-ICU personnel, not working in a department of their choice	
[55]	↑CS: older age ↑BO: younger age ↑STS: female	↓STS: organizational support	↑BO/↑STS: fear of COVID-19
[39]		↑STS: caring for women with a traumatic labor experience/ personal trauma/unresolved loss history	
[43]		↑CS: desire of the same career for their children, personal choice to work in this unit ↑BO: desire to leave the unit, traumatic event intensity ↓STS: staff works as a team, more years of working experience, physical health ↑STS: traumatic event intensity, desire to leave the unit	
[41]	↑CS: female, single, none or one child ↑STS: secondary education	↑CS: work experience >11 years, working in emergency departments ↓CS: working at pathological departments ↑BO/STS: working at pathological, emergency, and COVID-19 departments, job stress and its dimensions (workload, time pressure, occupational safety, bureaucratic procedures), clarity of objectives (task distribution and conflicting roles), business travel or meetings (high travel frequency and length of meetings)	
[47]		↑CS/↓BO: work engagement (vigor, dedication, absorption)	↑CS: psychological well-being, self-acceptance ↓STS: psychological well-being, environmental mastery
[42]			↑STS: moral distress
[31]			↑CS: perspective taking, empathic concern, mindfulness ↓CS: personal distress ↑BO: personal distress, self-judgement, isolation ↑STS: empathic concern, personal distress, self-judgement
[27]	↑CS: older age ↑STS: younger age, female, without leisure activities	↑STS: less job experience	
[32]			↑CS/↓STS/↓BO: empathic concern, perspective taking, self-compassion, psychological flexibility ↓CS: higher personal distress, psychological inflexibility ↑BO/↑STS: psychological inflexibility
[53]	↑CS: being parents ↑STS: female	↑BO: working at the pathological, emergency and COVID-19 units, job stress and its dimensions (characteristics of work: workload, time pressure, occupational safety, bureaucratic procedures), clarity of objectives (task distribution and conflicting roles)	
[35]	↑BO: age, marital status, number of children	↑BO: exposure to traumatic events/patients in intense suffering, palliative care group, workload (long working hours, shifts) ↓BO: supportive work environment/positive interdisciplinary team dynamics/ positive communication/stress management workshops	↓BO: personal skills (mindfulness, meditation, coping)
[44]			↑CS/↓BO/↓STS: resilience ↓CS/↑BO/↑STS: psychological distress (stress, anxiety, depression)
[28]	↓CS/↑BO/↑STS: female gender	↑CS: professional role, professional, seniority, positive attitude, problem orientation, transcendent orientation ↑BO/↑STS: avoidance strategies, problem orientation, transcendent orientation	↑CS/↑BO/↑STS: social support
[33]		↓CS/↑BO/↑STS: nursing profession	
[30]		↑CS/↓BO/↓STS: professional and care unit identification	↑BO/↑STS: emotional maladjustment
[46]		↑BO/↑STS: working overtime	↑CS: social support
[37]			↑CS: perspective taking, empathic concern ↓CS/↑BO: personal distress ↑STS: empathic concern, personal distress ↓BO: empathic concern
[26]	↓STS: higher education	↑CS/↓BO: longer experience	
[40]		↑CS: building/maintaining relationships with parents, exchanging information, sharing decision-making, having enough time for communication ↑BO: exchanging information, sharing decision ↓BO/↓STS: having enough time/ space for communication	

Note: CS: compassion satisfaction; BO: burnout; STS: secondary traumatic stress; ProQOL: professional quality of life.

## 4. Discussion

### 4.1. Summary of main findings

To our knowledge, this is the first systematic review to synthesize factors associated with professional quality of life among nurses working in European countries. By including only studies that used the ProQOL instrument and assessed all three of its dimensions—compassion satisfaction, burnout, and secondary traumatic stress—this review provides a comprehensive overview of individual, occupational, and psychosocial determinants of nurses' professional quality of life in the European context.

Thirty-five studies of moderate to high methodological quality were included, encompassing a total sample of 10,853 participants, the majority of whom were nurses working in hospital settings. Most studies reported moderate mean levels of compassion satisfaction and of the negative ProQOL dimensions, indicating that European nurses experience a complex balance between the rewards and costs of caregiving. At the same time, these findings should be interpreted in light of the uneven geographic distribution of the included studies, which were concentrated mainly in Southern Europe, with more limited representation from Northern and Central/Eastern Europe. As a result, the review offers a European perspective, but not an equally detailed picture of all European subregions. Demographic factors such as older age and, in several studies, female gender were generally associated with higher compassion satisfaction, whereas younger age, urban residence, and certain family responsibilities (e.g., parenting) were more often linked to higher burnout and secondary traumatic stress. In addition, the association between higher educational attainment and burnout in some studies may reflect unmet professional expectations, role strain, and a possible mismatch between nurses' competencies, level of training, and the scope of tasks, autonomy, or recognition available in their actual clinical roles.

Work-related factors formed a prominent cluster of determinants. High workload, frequent overtime, rotating shifts, temporary employment status, job stress, workplace violence, and employment in high-intensity settings—such as intensive care, emergency departments, COVID-19 units, oncology, palliative care, and primary care—were consistently associated with higher burnout and secondary traumatic stress. In contrast, greater work experience, clear professional roles, strong identification with one's unit, work engagement, lower work–family conflict, workload control, supportive team climates, and opportunities for training (e.g., in palliative care or coping with death) were linked to higher compassion satisfaction. Because the evidence base consisted predominantly of cross-sectional studies, these determinants should be interpreted as factors associated with ProQOL rather than as causal predictors.

Psychological and social factors also played a critical role. Resilience, psychological flexibility, self-compassion, self-acceptance, adaptive coping strategies, mindfulness, and robust social support networks (from colleagues, supervisors, family, and friends) were associated with higher compassion satisfaction and lower burnout and secondary traumatic stress. Conversely, neuroticism, chronic stress, personal distress, self-judgement, psychological inflexibility, fear related to the COVID-19 pandemic, moral distress, and social isolation were consistently linked to greater vulnerability to burnout and compassion fatigue. Overall, the findings suggest that nurses' professional quality of life in Europe is shaped by the interaction of demographic characteristics, demanding work environments, and the availability—or absence—of psychological and social resources.

### 4.2. Comparison with previous literature

The present findings are partly consistent with previous syntheses conducted in non-European or global samples. O'Brien-Pallas and Baumann (1992), as cited by Laserna Jiménez et al. (2022) [16], proposed a broad framework in which factors influencing nurses' professional quality of life are grouped into internal (e.g., individual, social, environmental, administrative) and external (e.g., patient demands, health policy, labor market) domains. Subsequent reviews have confirmed that professional quality of life can be conceptualized as an outcome or process shaped by individual emotions and perceptions, with key predictors including leadership, interpersonal relationships, work environment, demographic characteristics, workload, education, and autonomy [57],[58]. In line with this framework, our review highlights the central role of workload, work–family conflict, organizational climate, and social support—as well as individual psychological resources such as resilience and self-compassion—in shaping ProQOL among European nurses.

A systematic review of ten studies across five countries reported that approximately half of the included samples were satisfied with their professional quality of life, with satisfaction influenced by workload, autonomy, support, recognition, motivation, and intention to leave the profession [16]. Zhang et al. (2018) [22], in a meta-analysis of 21 studies, identified relatively high levels of compassion fatigue (52.6%) and burnout (52.0%), alongside elevated compassion satisfaction (47.6%), highlighting nurses' susceptibility to chronic stress and the coexistence of positive and negative professional experiences. Our review similarly found predominantly moderate levels of compassion satisfaction and of the negative ProQOL dimensions in European settings, suggesting that European nurses experience a comparable tension between fulfillment and strain to that described in global samples.

More recent reviews have focused on specific occupational stressors or practice areas. Galanis et al. (2024) [14] demonstrated that workplace bullying is negatively associated with compassion satisfaction and positively associated with burnout and secondary traumatic stress, underscoring the detrimental impact of toxic work environments. Lobo et al. (2024) [18] reported moderate levels of burnout, secondary traumatic stress, and job satisfaction among mental health nurses, with better outcomes among those with higher resilience, healthier lifestyles, and more supportive workplace conditions. These results are consistent with our finding that resilience, self-care, psychological flexibility, and social support operate as key protective factors.

Xie et al. (2021) [59] found that Asian countries showed lower compassion satisfaction and higher compassion fatigue compared with American and European settings, and that compassion fatigue increased between 2010 and 2019, peaking just before the COVID-19 pandemic. These global trends resonate with our observation of moderate levels of both positive and negative ProQOL dimensions among European nurses and highlight the influence of sociocultural and health-system context on ProQOL. Unlike previous global reviews, however, the present study specifically delineates these associations within European healthcare systems, where staffing models, labor conditions, and social policies may differ substantially from those in other regions. In this sense, our review adds nuanced, context-specific evidence on how demographic, work-related, and psychosocial factors converge to shape ProQOL in Europe.

### 4.3. Implications for practice and policy

The findings of this review have several implications for clinical practice, management, and health policy. First, the consistent association of high workload, shift work, overtime, job stress, workplace violence, and high-intensity clinical areas with burnout and secondary traumatic stress underscores the need for organizational interventions aimed at improving staffing levels, workload distribution, and scheduling practices. Ensuring adequate nurse–patient ratios, limiting excessive overtime, implementing fair and predictable shift patterns with schedules communicated in advance, protecting rest opportunities during shifts where feasible, and reducing avoidable bureaucratic demands may help to alleviate chronic stressors and protect nurses' professional quality of life. Hospital management may also consider routine monitoring of workload pressure, overtime burden, and staff rotation in high-intensity units, so that emerging risks for burnout and secondary traumatic stress can be identified earlier.

Second, the importance of organizational climate and social support suggests that interventions should also target team dynamics and leadership practices. Promoting supportive, non-punitive cultures, strengthening interdisciplinary collaboration, and training managers in transformational and compassionate leadership may reduce burnout and enhance compassion satisfaction. Programs that explicitly address workplace violence and bullying—through prevention policies, reporting mechanisms, and support for affected staff—are likewise critical. More specifically, hospital managers could implement clear reporting and follow-up procedures for violent incidents, regular team-based reflective meetings, and supervisor training focused on supportive communication, early recognition of distress, and appropriate referral of staff for support when needed.

Third, the strong influence of psychological and social resources indicates that individual-level interventions can complement organizational changes. Evidence from the included studies points to the potential value of training in resilience-building, mindfulness, psychological flexibility, self-compassion, and adaptive coping strategies. Structured self-care programs, peer support groups, supervision, and debriefing after traumatic events may further enhance nurses' capacity to manage emotional demands and sustain compassion satisfaction. In practical terms, this may include access to staff counselling or psychologist-led support services, facilitated peer-support groups, regular clinical supervision, and structured debriefing after particularly distressing clinical events.

**Figure 2.**
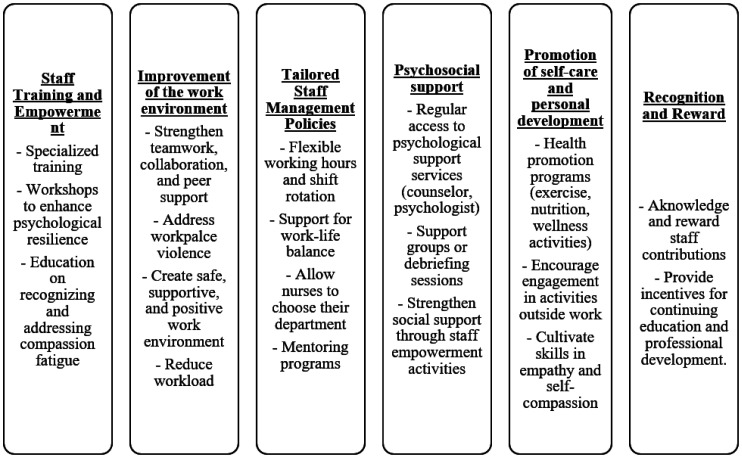
Proposed improvement interventions of nurses' ProQOL.

Finally, because many risk and protective factors operate simultaneously at individual, team, and organizational levels, multi-component interventions are likely to be more effective than isolated measures. Figure 2 summarizes a range of proposed interventions aimed at improving ProQOL among nurses, including strategies to optimize working conditions, strengthen social and managerial support, and foster psychological resources and self-care. Implementing and rigorously evaluating such interventions in diverse European settings should be a priority for healthcare organizations and policymakers seeking to promote nurses' well-being and retention.

### 4.4. Implications for research

This review also highlights important directions for future research. The concentration of available evidence in a limited number of European countries, predominantly in Southern and Western Europe, indicates the need for studies in underrepresented regions, including Central and Eastern Europe and the Nordic countries. Moreover, most included studies focused on hospital settings and used convenience samples, which limits generalizability. Future research should therefore extend to primary care, community health, home care, and long-term care settings and employ more robust sampling strategies.

Methodologically, the evidence base was dominated by cross-sectional designs, which preclude causal inference and do not allow the temporal direction of the observed associations between determinants and ProQOL to be established. Longitudinal studies are needed to clarify temporal and causal pathways, for example, whether changes in workload, leadership, or resilience precede changes in compassion satisfaction, burnout, and secondary traumatic stress. Intervention studies should evaluate the effectiveness and sustainability of multi-level programs designed to enhance ProQOL, with particular attention to high-risk groups such as younger nurses, those working in high-acuity or understaffed units, and those exposed to workplace violence or moral distress.

Finally, future research could explore how broader health-system and policy-level factors—such as national staffing regulations, occupational health programs, and labor market conditions—interact with individual and organizational determinants to influence nurses' professional quality of life. Such work would support the development of comprehensive strategies that integrate clinical, organizational, and policy perspectives to protect and promote ProQOL across European healthcare systems.

## 5. Limitations

There are several limitations in this systematic review. First, the evidence base consisted predominantly of cross-sectional observational studies, with only very limited longitudinal evidence. Therefore, the review identifies associations between examined factors and the three ProQOL dimensions, but it does not support causal inferences or firm conclusions about temporal directionality. Second, professional quality of life was assessed exclusively through self-report measures (ProQOL), which may be subject to recall bias, social desirability bias, and common method variance. The reliance on a single type of instrument, although it enhances comparability across studies, may also limit the scope of constructs examined and does not capture objective indicators of occupational stress or well-being. Third, the majority of the included studies recruited nurses working in hospital settings and used convenience sampling. As a result, the generalizability of our findings to nurses working in primary care, community health, home care, and long-term care is limited, and external validity is reduced. In addition, most studies were conducted in a relatively small number of European countries, mainly in Southern and Western Europe. Furthermore, 13 included studies were conducted during the coronavirus disease period in 2019, which may have influenced both the reported levels of professional quality of life and the observed associations with occupational and psychosocial factors. As a result, part of the evidence base may reflect pandemic-specific working conditions rather than routine clinical contexts. Fourth, substantial heterogeneity in study populations, settings, measures of exposure, and analytic approaches did not allow for a formal meta-analysis or quantitative pooling of effect sizes. Instead, we adopted a narrative synthesis, which, although appropriate in this context, may be more vulnerable to subjective interpretation. Finally, we restricted inclusion to articles published in English and to studies using the ProQOL instrument, which may have introduced language and publication bias, and may have excluded relevant evidence based on other measures of occupational well-being.

## 6. Conclusions

This systematic review identified a broad set of demographic, work-related, and psychological/social factors that influence nurses' professional quality of life in European settings. Overall, younger age, adverse working conditions, high workload, shift work, overtime, workplace violence, job stress, lack of organizational support, low social support, maladaptive coping strategies, and maladaptive psychological traits (such as neuroticism, psychological inflexibility, and self-judgement) were consistently associated with higher burnout and secondary traumatic stress. In contrast, older age, greater work experience, specialized training (e.g., in palliative care and coping with death), clear professional roles, work engagement, lower work–family conflict, positive organizational climates, resilience, self-compassion, psychological flexibility, effective self-care, mindfulness, and strong social support networks were associated with higher compassion satisfaction and better overall ProQOL.

These findings underscore the need for multi-level strategies to protect and promote nurses' professional quality of life in Europe. At the organizational level, improving staffing levels, workload distribution, scheduling practices, leadership styles, team functioning, and workplace safety is critical to reducing occupational stressors and preventing burnout and secondary traumatic stress. At the individual level, interventions that strengthen resilience, self-compassion, psychological flexibility, mindfulness, and adaptive coping, as well as structured self-care and peer support programs, may help nurses to sustain compassion satisfaction despite demanding working conditions.

Ultimately, safeguarding nurses' professional quality of life is essential not only for their mental health, retention, and job satisfaction but also for the sustainability, quality, and safety of healthcare services across hospitals, primary care, community health, and long-term care. Investing in policies and interventions that address both structural work factors and individual psychological resources can contribute to a more resilient nursing workforce and to improved outcomes for patients, professionals, and health systems in the European context.

## Use of AI tools declaration

The authors declare they have not used Artificial Intelligence (AI) tools in the creation of this article.


