# Supplementary material for: Factors affecting nurses' professional quality of life in Europe - A systematic review
Source: AIMS Public Health. 2026 Apr 16;13(2):485–512. doi: 10.3934/publichealth.2026026 (PMC13368625; doi:10.3934/publichealth.2026026)
Supplement: Supplementary file 1 [file publichealth-13-02-026-s001.pdf]

Public Health

AIMS Public Health, 13(2): 485–512.
DOI: 10.3934/publichealth.2026026
Received: 05 January 2026
Revised: 26 March 2026
Accepted: 31 March 2026
Published: 16 April 2026

http://www.aimspress.com/journal/aimsph

Review

Factors affecting nurses' professional quality of life in Europe - A systematic review

Efrosini Vera[1,*], Petros Galanis[1,2], Polyxeni Mangoulia[1], and Theodoros Pesiridis[1,3]

[1] Faculty of Nursing, National and Kapodistrian University of Athens, 123 Papadiamantopoulou Str., Athens, 11527, Greece
[2] Clinical Epidemiology Laboratory, Faculty of Nursing, National and Kapodistrian University of Athens, 123 Papadiamantopoulou Str., Athens, 11527, Greece
[3] Community Nursing Laboratory, Faculty of Nursing, National and Kapodistrian University of Athens, 123 Papadiamantopoulou Str., Athens, 11527, Greece

* **Correspondence:** Email: efvera@nurs.uoa.gr; Tel: +302121040188.

Supplementary

Table S1. Results of JBI appraisal tools for cross-sectional studies.

Reference	1. Were the criteria for inclusion in the sample clearly defined?	2. Were the study subjects and the setting described in detail?	3. Was the exposure measured in a valid and reliable way?	4. Were objective, standard criteria used for measurement of the condition?	5. Were confounding factors identified?	6. Were strategies to deal with confounding factors stated?	7. Were the outcomes measured in a valid and reliable way?	8. Was appropriate statistical analysis used?	Total quality
Sansó et al. (2015) [51]	YES	YES	YES	YES	YES	YES	YES	YES	High
Arimon-Pagès et al. (2019) [4]	YES	YES	YES	YES	YES	YES	YES	YES	High
Ruiz-Fernández et al. (2020) [1]	YES	YES	YES	YES	YES	YES	YES	YES	High
Arribas-García et al. (2020) [25]	YES	YES	YES	YES	YES	YES	YES	YES	High
Ruiz-Fernández et al. (2021) [48]	YES	YES	YES	YES	YES	YES	YES	YES	High
Sansó et al. (2020) [51]	YES	YES	YES	YES	YES	YES	YES	YES	High
Ruiz-Fernández et al. (2021) [49]	YES	YES	YES	YES	YES	YES	YES	YES	High
Sarabia-Cobo et al. (2021) [52]	YES	YES	YES	YES	YES	YES	YES	YES	High
Galiana et al. (2022) [34]	YES	YES	YES	YES	YES	YES	YES	YES	High
Pérez-Ardanaz et al. (2022) [45]	YES	YES	PARTIALLY	YES	YES	UNCLEAR	YES	YES	High
González-Nuevo et al. (2024) [36]	YES	YES	YES	YES	YES	YES	YES	YES	High
Almansa-Sáez et	YES	YES	YES	YES	YES	YES	YES	YES	High

al. (2024) [24]									
Mangoulia et al. (2015) [7]	YES	YES	YES	YES	YES	YES	YES	YES	High
Skoufi et al. (2018) [54]	YES	YES	YES	YES	YES	YES	YES	YES	High
Kartsonaki et al. (2023) [38]	YES	YES	YES	YES	YES	YES	YES	YES	High
Stefanatou et al. (2022) [55]	YES	YES	YES	YES	YES	YES	YES	YES	High
Katsantoni et al. (2019) [39]	YES	YES	YES	YES	YES	PARTIALLY	YES	YES	High
Mavratza et al. (2021) [43]	YES	YES	YES	YES	YES	YES	YES	YES	High
Latsou et al. (2022) [41]	YES	YES	YES	YES	YES	YES	YES	YES	High
Rikos et al. (2024) [47]	YES	YES	YES	YES	YES	YES	YES	YES	High
Malliarou et al. (2021) [42]	YES	YES	YES	YES	YES	YES	YES	YES	High
Duarte et al. (2016) [31]	YES	YES	YES	YES	PARTIALLY	PARTIALLY	YES	YES	High
Borges et al. (2019) [27]	YES	YES	YES	YES	YES	YES	YES	YES	High
Duarte et al. (2017) [32]	YES	YES	YES	YES	PARTIALLY	PARTIALLY	YES	YES	High
Serrao et al. (2022)	YES	YES	YES	YES	YES	UNCLEAR	YES	YES	High
Goncalves et al.	YES	YES	YES	YES	YES	YES	YES	YES	High

(2025)									
Morando et al. (2024) [44]	YES	YES	YES	YES	PARTIALLY	PARTIALLY	YES	YES	High
Calegari et al. (2022) [28]	YES	YES	YES	YES	YES	YES	YES	YES	High
Franza et al. (2020) [33]	YES	YES	YES	YES	PARTIALLY	NO	YES	PARTIALLY	Moderate
Caricati et al. (2023) [30]	YES	YES	YES	YES	YES	YES	YES	YES	High
Pergol-Metko et al. (2023) [46]	YES	YES	YES	YES	PARTIALLY	NO	YES	YES	High
Hunt et al. (2019) [37]	YES	YES	YES	YES	YES	YES	YES	YES	High
Blixt et al. (2023) [26]	YES	YES	YES	YES	PARTIALLY	YES	YES	YES	High
Labrie et al. (2024) [40]	YES	YES	YES	YES	YES	YES	YES	YES	High

AIMS Press

© 2026 the Author(s), licensee AIMS Press. This is an open access article distributed under the terms of the Creative Commons Attribution License (http://creativecommons.org/licenses/by/4.0)
